# Atypical Mitochondrial Phenotype of Colonic CD4+ and CD8+ T Cells During Experimental Chronic Colitis

**DOI:** 10.3390/biomedicines13092094

**Published:** 2025-08-28

**Authors:** Jonas Negele, Tobias Franz, Anna Krone, Marc Roder, Elif Gelmez, Nouria Jantz-Naeem, Olivia Kershaw, Verena Keitel-Anselmino, Andreas Jeron, Dunja Bruder, Sascha Kahlfuß

**Affiliations:** 1 Institute of Molecular and Clinical Immunology, Medical Faculty, Otto-von-Guericke-University Magdeburg, 39120 Magdeburg, Germany; j-negele@gmx.net (J.N.); tobi.franz@mailbox.org (T.F.); anna.krone@med.ovgu.de (A.K.); marc.roder@st.ovgu.de (M.R.); nouria.jantz-naeem@med.ovgu.de (N.J.-N.); 2 Research Group Infection Immunology, Institute of Medical Microbiology and Hospital Hygiene, Otto-von-Guericke-University Magdeburg, 39120 Magdeburg, Germany; elif.gelmez11@gmail.com (E.G.); andreas.jeron@helmholtz-hzi.de (A.J.); dunja.bruder@med.ovgu.de (D.B.); 3 Department of Veterinary Medicine, Institute of Veterinary Pathology, Freie Universität Berlin, 14163 Berlin, Germany; olivia.kershaw@fu-berlin.de; 4 Department of Gastroenterology, Hepatology and Infectious Diseases, University Hospital of Magdeburg, 39120 Magdeburg, Germany; verena.keitel-anselmino@med.ovgu.de; 5 Health Campus Immunology, Infectiology and Inflammation (GCI^3^), Medical Faculty, Otto-von-Guericke-University Magdeburg, 39120 Magdeburg, Germany; 6 Research Group Immune Regulation, Helmholtz Centre for Infection Research, 38124 Braunschweig, Germany; 7 Institute of Medical Microbiology and Hospital Hygiene, Medical Faculty, Otto-von-Guericke-University Magdeburg, 39120 Magdeburg, Germany; 8 Center for Health and Medical Prevention (CHaMP), Otto-von-Guericke-University Magdeburg, 39120 Magdeburg, Germany

**Keywords:** ulcerative colitis, inflammatory bowel disease, t cells, b cells, neutrophils, intestinal epithelial cells, DSS colitis, autoimmune colitis, metabolism

## Abstract

**Background:** Patients with ulcerative colitis (UC) suffer from a chronic relapsing-remitting inflammatory bowel disease and show heterogeneous disease severity and therapy response. It was recently reported that rectal biopsies from patients with UC show a downregulation of various mitochondrial genes during active UC. **Methods:** We used dextran sulfate sodium (DSS)-induced colitis as a preclinical mouse model for UC to study metabolic characteristics of ex vivo colonic lamina propria CD4+ and CD8+ T cells, B cells, eosinophils, neutrophils and intestinal epithelial cells during experimental acute and chronic inflammatory bowel disease and remission state. **Results:** Our results indicate that CD4+ and CD8+ T cells in the colon produce significantly less mitochondrial reactive oxygen species and possess smaller mitochondria during chronic DSS-induced colitis, which is resolved during remission state. In addition, CD4+ and CD8+ T cells exhibit increased glucose uptake during acute but defective glucose consumption during chronic DSS colitis. **Conclusions:** Together, our data provide evidence for an atypical mitochondrial phenotype of colonic CD4+ and CD8+ T cells during chronic colitis that is resolved during the remission phase of the disease.

## 1. Introduction

Inflammatory bowel disease (IBD), which includes ulcerative colitis (UC) and Crohn’s disease (CD), is characterized by chronic, progressive inflammation of the bowel leading to a variety of digestive and systemic symptoms, including weight loss, diarrhea, intestinal bleeding anemia and fatigue [1,2,3]. IBD is associated with complex interactions between genetic and environmental risk factors that alter the gut microbiome and promote mucosal immune dysregulation [4,5,6]. Multiple genetic regions linked to the development of inflammatory bowel disease have been discovered promoting barrier maladaptation [7], including variations in the melanocortin system [8]. CD can affect the entire bowel in a discontinuous transmural pattern, whereas UC mainly affects the mucosa and submucosal layer of the rectum and colon in a continuous pattern [9]. IBD is further characterized by a barrier dysfunction of intestinal epithelial cells (IECs), which sense bacteria and can initiate appropriate defense mechanisms in healthy individuals [10]. Dysfunctional IECs and leaky intestinal barriers lead to activation of cells residing in the lamina propria and the production of cytokines that promote the invasion of adaptive and innate immune cells [9]. Importantly, disruption of the complex cytokine and mediator signatures that mediate communication between the epithelium and both the innate and adaptive immune systems promotes and perpetuates intestinal inflammation, leading to intestinal fibrosis, abscesses, ulceration and epithelial hyperplasia [9,10]. Among the most significant immune cells involved in the pathogenesis of IBD are CD4+ and CD8+ T cells, B cells, eosinophils and neutrophils [11]. Here, CD is mainly characterized by a CD4+ T helper (Th)1- and Th17-dependent pathophysiology involving the cytokines IL-12 and IL-23. In contrast, Th2 cells and their cell-specific cytokines IL-4, IL-5 and IL-13, as well as IL-9, amplify and maintain UC [12,13].

Recently it became evident that during IBD, immunometabolism is involved in the pathogenesis of the disease [14]. The onset of intestinal inflammation is closely interlinked to the imbalanced ratio between controlling regulatory T cells (Tregs) and proinflammatory Th cells. During IBD, colitogenic T cells display mitochondrial dysfunction and an altered immunometabolism including reduced mitochondrial respiration, upregulated glutamine metabolism and increased lactate production [15]. Additionally, dietary and microbiota-derived factors significantly influence T cell metabolism in the colon, impacting immune responses and disease pathogenesis [16]. Specific nutrients, such as short-chain fatty acids (SCFAs), produced by gut bacteria during the fermentation of dietary fiber, or bile acids, affect T cell differentiation and function [17,18,19,20,21]. Short-chain fatty acids (SCFAs) promote the differentiation of CD4+ T cells into regulatory T cells (Tregs) while inhibiting Th17 cell development in the colon by inducing histone acetylation through inhibition of histone deacetylases (HDACs), which enhances Foxp3 expression and suppresses RORγt [22]. Conversely, dietary choices can also modulate the gut microbiota, which in turn influences T cell behavior. This indicates an important relationship between immunometabolism and gut inflammation. Further, the arising intestinal inflammation also seems to drive metabolic alterations within the colonic tissue. This was demonstrated by a recent study by Haberman and colleagues [23]. The latter study showed, especially in active disease, a significant reduction in mitochondrial gene expression and mitochondrial function in rectal biopsies of UC patients, likely indicating a specific disruption of mitochondrial energy production. Specifically, in CD45+ leukocytes, the mitochondrial membrane potential, indicative of the cellular capacity for ATP production, as well as the expression of *PPARGC1A* (PGC-1α), the master regulator of mitochondrial biogenesis, were reduced in UC patients. The downregulation of mitochondrial gene expression is specific for colon biopsies from UC patients, suggesting mitochondrial dysfunction plays a pivotal role in the pathogenesis of UC. Further, besides mitochondrial metabolism, during experimental DSS-induced IBD in vivo, the enzyme ATP-citrate lyase (ACLY), connecting glucose and lipid metabolism, mediates inflammation as its activity is critical for the cytokine production of mucosal T cells [24]. These findings indicate that T cell immunometabolism, its adjustments during inflammation and its sensitive manipulation are critical during the pathogenesis of IBD. However, the underlying mechanisms remain incompletely understood. In addition, there is a lack of appropriate experimental models to systematically investigate the underlying mechanisms in more detail.

Here, we utilized a mouse model of DSS-induced colitis mimicking several key features of human IBD to investigate metabolic characteristics of colonic CD4+ and CD8+ T cells, B cells, eosinophils, neutrophils and intestinal epithelial cells during acute and chronic IBD-like disease and remission. Acute DSS-induced colitis is induced by short-term administration of DSS, leading to a rapid onset of inflammation, whereas in chronic DSS-induced colitis, repeated cycles of DSS administration with recovery periods in between lead to persistent inflammation and tissue remodeling [25]. In addition, the acute model is primarily associated with tissue damage, while the chronic model is characterized by significant fibrosis and scarring due to ongoing tissue repair processes [25].These models resemble the different phases of disease progression of IBD in humans, and understanding these differences is crucial for studying different aspects of IBD pathogenesis.

Our results show that during chronic DSS-induced colitis, colon CD4+ and CD8+ T cells produce fewer mitochondrial reactive oxygen species (mROS) and have reduced-mitochondria sizes; this phenotype is only transient and normalizes during the remission phase of the disease. Moreover, while CD4+ and CD8+ T cells show increased glucose uptake in acute DSS colitis, they display impaired glucose consumption in its chronic phase, a condition that persists until the remission phase. Together, our data provide evidence of an atypical mitochondrial phenotype of colonic CD4+ and CD8+ T cells during chronic colitis that returns to the initial state during the remission phase of the disease.

## 2. Materials and Methods

### 2.1. Mice, Colitis Induction and Disease Scoring

All animal experiments were conducted using female C57BL/6JRj mice (Janvier Labs, 22 Rte des Chenes Secs, 53940 Le Genest-Saint-Isle, France), which were 11 weeks old at the start of the experiment. All animals were sourced from the same breeding facility. The mice were kept in a specific pathogen-free environment at the central animal facility of the Medical Faculty, Otto-von-Guericke-University, Magdeburg. Mice were sacrificed by CO_2_ inhalation. Anesthesia was not performed. Acute colitis was induced by providing 2% (*w*/*v*) DSS (36,000–50,000 Da, MP Biomedicals (3 Hutton Centre Drive Suite 100, Santa Ana, CA 92707, USA) in the drinking water ad libitum for six days. To facilitate recovery from acute colitis, the mice were subsequently given DSS-free drinking water for 19 days. Sampling was conducted on day 6 for the acute group, day 10 for the young control group, and day 25 for the acute remission group following induction. Chronic colitis was induced through three successive cycles of DSS (1.7% *w*/*v*), each lasting six days with ad libitum access, followed by a 14-day recovery period with DSS-free drinking water after the first and second cycle. A remission phase from chronic colitis was achieved by supplying regular drinking water for 21 days after the final DSS cycle. For the chronic group, sampling occurred on day 46; for the old control group, on day 52; and for the chronic remission group, on day 67 after induction. Control mice were maintained on regular drinking water throughout the study. Relative body weight loss was measured, and the disease activity index (DAI) was calculated based on body weight loss, stool consistency and the presence of blood in feces. Occult blood in fecal samples was assessed using the hemoCARE assay (CARE diagnostica, Voerde, Germany).

### 2.2. Histopathology of Colon

Following euthanasia, the colons and cecums were placed in histology cassettes, fixed in 4% PFA and subsequently embedded in paraffin. Sections 5 µm in size were dewaxed and stained with hematoxylin and eosin (HE). Histopathological scoring was conducted using the following parameters: immune cell infiltration, epithelial damage, extent of tissue damage and the proportion of tissue affected by inflammation. The specific scoring criteria are detailed in Table 1.

### 2.3. Isolation of Lamina Propria Leukocytes from Colon

Colonic lamina propria leukocytes were extracted following the protocol provided with the Lamina Propria Dissociation Kit from Miltenyi Biotec (Bergisch-Gladbach, Germany). The samples were incubated in a 37 °C shaker incubator. Total leukocytes were isolated from the digested tissue suspension using Percoll (GE Healthcare, Uppsala, Sweden) density gradient centrifugation (~1.041 g/mL) for 20 min at 1800 rpm at room temperature, with the rotor brake turned off. After centrifugation, the supernatant was removed, and the leukocyte pellet was rinsed once with phosphate-buffered saline (PBS). Half of the isolated leukocyte suspension was reserved for fluorescent activated cell sorting (FACS) analysis.

### 2.4. Flow Cytometry

For identifying and characterizing different cell subsets, the isolated cell suspension was stained with fluorochrome-labeled antibody panels and analyzed by flow cytometry. The panel comprised CD45 (BioLegend, San Diego, CA, USA, # 103135), CD4 (BioLegend, # 100516 or # 100423) and CD8 (BioLegend, # 100722), MHCII (BioLegend, # 107636 or # 17620), Siglec-F (BioLegend, # 155507 or # 155504), Ly6G (BioLegend, # 127606 or # 127612), B220 (BioLegend, # 103205 or # 103211) and Ep-CAM (BioLegend, # 118225). Epithelial cells were gated as CD45-CD31-Ep-CAM+ cells, B cells were gated as CD45+B220+MHCII+ cells and CD4+ and CD8+ T cells were gated as CD45+CD4+ and CD45+CD8+, respectively. Eosinophils were gated as CD45+SSC^high^Siglec-F+ cells. Neutrophils were gates as CD45+Ly6G+ cells.

### 2.5. Uptake of Glucose and Amino Acids

Glucose uptake was assessed using the fluorescent glucose analog 2-NBDG (Thermo Fisher Scientific, Waltham, MA, USA, # N13195). Following isolation, the cells were incubated in glucose-free RPMI medium with 100 μM 2-NBDG for 90 min in the dark. The quantity of 2-NBDG taken up by the cells was measured using FACS after surface staining. Amino acid uptake was evaluated through flow cytometry with the Amino Acid Uptake Assay Kit (Dojindo Laboratories, Munich, Germany, # UP04-12). After isolation, the cells were incubated in amino acid-free DMEM medium (PAN-Biotech, Aidenbach, Germany, # P04-01507) containing the amino acid analog BPA for 30 min in the dark (BPA uptake solution). After two washing steps, the cells were treated for 10 min with a working solution that includes a fluorescent probe capable of crossing the cell membrane, binding to intracellularly accumulated BPA, and emitting fluorescence (λ_ex_ = 360 nm, λ_em_ = 460 nm).

### 2.6. Measurement of mROS Production and Mitochondrial Volume

Following isolation, mitochondrial ROS production was assessed by incubating the cells with 5 μM MitoSOX™ Red Mitochondrial Superoxide Indicator (Thermo Fisher Scientific, #M36008) for 10 min in PBS containing 2% FBS, protected from light. To measure mitochondrial volume via FACS, cells were stained with MitoTracker Red FM FM (Thermo Fisher Scientific, # M7514) for 30 min under similar conditions (PBS with 2% FBS, in the dark).

### 2.7. Expression of GLUT1, GLUT3 and CD36

Upon isolation and staining for cell-specific surface markers, we analyzed Glut-1 and Glut-3 expression. Cells were stained with Glut-1 (Thermo Fisher Scientific, # MA5-31960) or Glut-3 (Thermo Fisher Scientific, # MA5-32697) in PBS for 30 min on ice after incubation with Fc-block (Thermo Fisher Scientific, # 14-0161-86). After washing, cells were incubated with goat anti-rabbit antibody (Thermo Fisher Scientific, # A32731) and analyzed using flow cytometry. For CD36 staining, we used cell-specific markers and anti-CD36-APC (BD Bioscience, Temse, Belgium # 562744). All of these experiments were conducted using FMOs as control.

### 2.8. Re-Analysis of Previously Published RNA-Seq Data

We used the 5296 significantly altered genes (|fold change| ≥ 1.5, FDR < 0.001) from the comparison of ulcerative colitis (UC) (206 patients) and non-IBD controls (20 patients) stated in supplementary data 1 of [23]. The samples were isolated from human rectal biopsies obtained during diagnostic colonoscopy. Resulting gene symbols were analyzed for over-represented terms from the Kyoto Encyclopedia of Genes and Genomes (KEGG) and the Gene Ontology (GO) consortium using DAVID Bioinformatics online tool (https://davidbioinformatics.nih.gov/home.jsp, accessed on 19 August 2023) focusing on metabolic terms. Terms with *p*_adj_ < 0.05 were considered significantly over-represented.

### 2.9. T Cell Culture and Oligomycin Treatment

CD4+ T cells were isolated from the spleen and cervical, submandibular, brachial, axillary, inguinal and mesenterial lymph nodes of WT mice by negative enrichment using the MagniSort Mouse CD4+ T Cell Enrichment Kit (Thermo Fisher Scientific, # 8804-6821-74). The culture plates were coated with rabbit anti-hamster IgG (Jackson Immuno Research, West Grove, PA, USA, # 307-005-003) for 2 h at 37 °C. CD4+ T cells were maintained in RPMI medium containing 10% FCS, 2 mM GlutaMAX (Thermo Fisher Scientific, # 35050038), 50 µM β-mercaptoethanol (Thermo Fisher Scientific, # 31350010), 2% penicillin/streptavidin (Thermo Fisher Scientific, # 15070063) and stimulated with 1 μg/mL anti-CD3ε and 1 μg/mL, as well as anti-CD28 antibodies (BioLegend, # 100340 and 102116). The cells were treated for 72 h in the presence or absence of 3 μM oligomycin (Agilent, Waldbronn, Germany, # 103010-100).

### 2.10. Statistical Analysis

All data were analyzed using GraphPad Prism 8.0 (GraphPad software, Inc., La Jolla, CA, USA) and are presented as the mean ± SEM, if not indicated otherwise. Comparisons between two groups were analyzed using the unpaired Student’s t test, while comparisons across multiple groups (murine DSS-induced colitis models) were performed using ordinary Kruskal–Wallis test (H-test), as the values were non-parametric. *p* values  <  0.05 were considered as statistically significant; different levels of significance were indicated as follows: * *p*  <  0.05; ** *p*  <  0.01; *** *p*  <  0.001, **** *p*  <  0.0001.

## 3. Results

### 3.1. Ulcerative Colitis Is Characterized by a Suppression of Mitochondrial Gene Expression

Patients with UC suffer from a chronic relapsing-remitting IBD and show a heterogeneous disease severity and therapy response. However, part of the affected patients does not respond to pharmacological therapy, which ultimately requires surgical resection of the inflamed bowel. The latter emphasizes that the pathogenesis of UC and its underlying disease endotypes are still incompletely understood. To date, only a few studies addressed the question whether IBD is associated with changes in the immunometabolism of the immune and non-immune cells involved in the disease pathogenesis and how sustainable potential metabolic changes manifest at the cellular level. In this context, it was recently reported that rectal biopsies from patients with UC show a significant suppression of mitochondrial gene expression during active UC [23].

**Figure 1:**
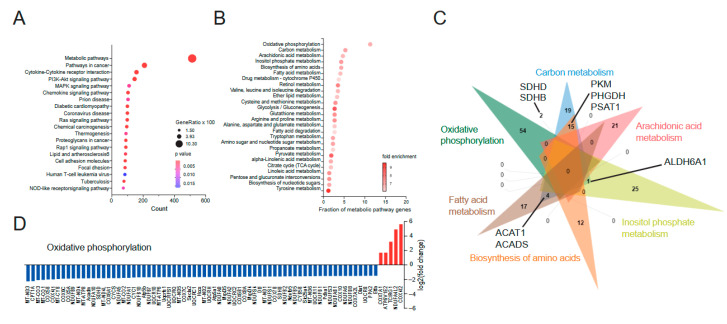
Ulcerative colitis is characterized by a suppression of mitochondrial genes. (**A**). Identification of altered pathways in human rectal UC biopsies by KEGG pathway term enrichment analysis. Data derives from [23] and was reanalyzed using 5296 significantly altered genes (|fold change| ≥ 1.5, FDR < 0.001) from the comparison of ulcerative colitis (UC) (206 patients) and non-IBD controls (20 patients) stated in supplementary data 1 of [23]. Terms with *p*_adj_ < 0.05 were considered significantly over-represented. The *x*-axis represents the number of over-represented gene symbols from the input data within stated KEGG terms. Circle size represents the term’s gene ratio (the percent of gene symbols from input data in reference to all gene symbols annotated in a given KEGG term). Color-coding of circles represents the significance of term enrichment (*p*-value). (**B**). KEGG metabolic pathway term enrichment analysis of over-represented genes from the ‘Metabolic pathway’ term from (**A**). *X*-axis represents the gene ratio of stated KEGG terms. Color-coding indicates fold-enrichment of over-representation. (**C**). Venn diagram of gene symbols annotated in the top six-fold-enriched metabolic pathway terms of (**B**). Selected gene symbols annotated within indicated pathways are stated. (**D**). log2 fold change of up (red)- and downregulated (blue) genes from the KEGG pathway term “Oxidative phosphorylation”, comparing UC patients with age- and sex-matched non-IBD controls [23], identified in (**B**). (**A**–**D**). Original RNA-seq data used for re-analyses were published in [23].

We reanalyzed RNA sequencing data from “The Predicting Response to Standardized Pediatric Colitis Therapy” (PROTECT) study [23] using KEGG term pathway analyses and found that most of the differentially expressed genes (DEGs) belong to the category “Metabolic pathways” (Figure 1A, KEGG ID: map01100). Most of the DEGs within these “Metabolic pathways” encoded for proteins are directly or indirectly involved in “Oxidative phosphorylation” (KEGG ID: hsa00190) with no overlap with other metabolic pathways (Figure 1B,C). For most of the genes within the “Oxidative phosphorylation” pathway, we found a significant downregulation in rectal biopsies from UC patients compared to non-IBD controls. Among the most downregulated genes were several nuclear-encoded genes of the electron transport chain (*ETC*) but also of metabolic key enzymes, including the rate-limiting enzyme of beta-oxidation, Carnitine palmitoyltransferase 1A (*CPT1A*) (Figure 1D). Together, these results indicate that rectal biopsies from patients with active UC show a significant downregulation of mitochondrial genes.

### 3.2. Dextran Sulfate Sodium-Induced Colitis as a Preclinical Mouse Model to Study Metabolic Characteristics in Colon Immune Cells

**Figure 2:**
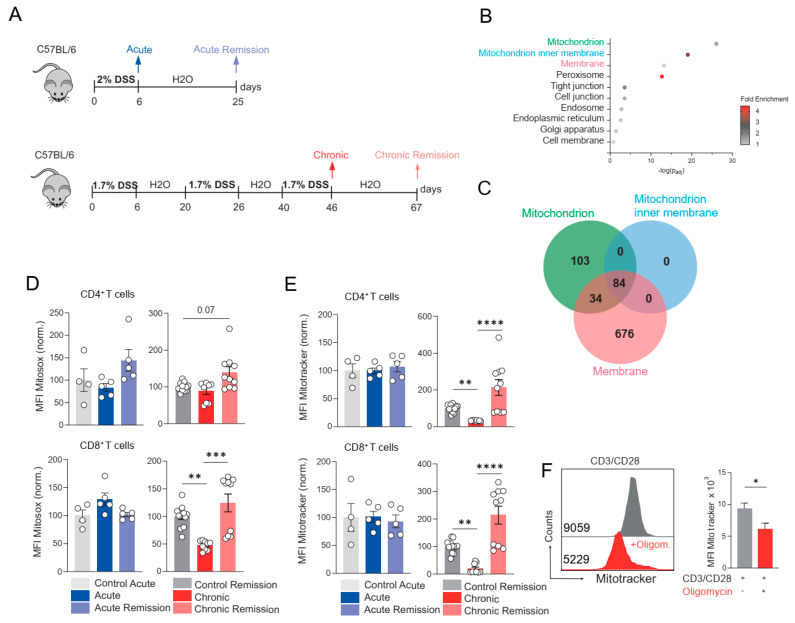
DSS-induced colitis results in decreased mitochondrial function within T cells. (**A**). Experimental setup for the induction of acute and chronic DSS-induced colitis in C57/BL6 mice. Acute model: Mice receive drinking water infused by 2% DSS for 6 consecutive days following a recovery period of 19 days with normal drinking water. Mice are sacrificed at day 6 (acute) and day 25 (remission of acute). Chronic model: To induce chronic colitis, WT mice received three cycles of 1.7% DSS, respectively, including a 14-day recovery period with drinking water after the first and second treatment cycles (day 46). Mice reach the stage of chronic remission by receiving normal drinking water for further 21 days after the third DSS treatment (day 67). (**B**). GO Biological pathway analysis of human rectal biopsies of UC patients using 5296 significantly altered genes (|fold change| ≥ 1.5, FDR < 0.001) from the comparison of ulcerative colitis (UC) (206 patients) and non-IBD controls (20 patients) stated in supplementary data 1 of [23]. Terms with *p*_adj_ < 0.05 were considered significantly over-represented. Color-coding represents fold enrichment of statistical over-representation of indicated pathway terms. *X*-axis shows −log_10_ (adjusted *p* value). Terms colored in green, blue, and red represent the top three over-represented terms. (**C**). Venn diagram of the gene symbol annotations using the top three over-represented terms from (**B**). (**D**,**E**) *Lamina propria* leukocytes from mouse colons from indicated experimental groups were isolated using enzymatic tissue digestion followed by density-gradient centrifugation. Mice in the six different experimental groups of the DSS model were treated and sacrificed as denoted in (**A**). (**D**). Mitochondrial ROS production was determined by Mitosox staining, followed by surface staining with lineage antibodies and subsequent flow cytometric analysis. CD4^+^ and CD8^+^ T cells were analyzed for their Mitosox mean fluorescence intensity (MFI). (**E**). Mitochondrial volume of CD4+ and CD8+ T cells was determined by Mitotracker staining and FACS analyses evaluating the Mitotracker MFI at indicated stages of DSS-induced colitis. (**F**). Mixed leukocytes from spleen and lymph nodes (cervical, submandibular, brachial, axillary, inguinal, mesenteric) were isolated from untreated wild-type mice, and CD4+ T cells were isolated using magnetic negative selection. Cells were stimulated by agonistic CD3/CD28 (1 µg/mL each) antibodies in the absence and presence of 3 µM Oligomycin for three days. Mitochondrial volume was determined by Mitotracker staining and FACS, analyzing Mitotracker MFI. Left: Representative histogram overlay. Numbers indicate MFI of histogram data. Right: Barplot showing mean ± SEM. Data in (**D**,**E**) of young control animals (*n* = 4); the stages of acute colitis (*n* = 5) and remission of acute colitis (*n* = 5) are from one experiment (N = 1). Data from old control animals (*n* = 12); the stages of chronic colitis (*n* = 11) and remission of chronic colitis (*n* = 11) are from two experiments (N = 2). Dots represent individual mice used to prepare cell solutions of the indicated cell subset. Bars and whiskers represent mean ± SEM. Statistical analysis in (**D**,**E**) was performed using the Kruskal–Wallis test (H-Test), using values normalized to the mean of control animals. ** *p*  <  0.01, *** *p*  <  0.001, **** *p* < 0.0001. Data in (**F**) is from one experiment (N = 1) using three individual mice (*n* = 3) to isolate and stimulate naïve CD4+ T cells. Statistical analysis in (**F**) was conducted using the unpaired Student’s *t*-test. * *p*  <  0.05.

To study the metabolic characteristics of adaptive and innate immune cells during IBD, we used a mouse model of dextran sulfate sodium (DSS)-induced colitis [26]. We induced acute colitis in wild-type (WT) C57BL/6JRj mice by administering 2% dextran sulfate sodium (DSS) in the drinking water ad libitum for 6 consecutive days, followed by a remission period of 19 days (Figure 2A). A subset of WT mice was sacrificed on day 6 to assess disease severity and adaptive and innate immune cells in the colon during acute DSS colitis (acute colitis), while another cohort was analyzed on day 25 during the remission phase (acute remission). To induce chronic colitis, WT mice received three cycles of 1.7% DSS, respectively, including a 14-day recovery period with drinking water after the first and second treatment cycles (Figure 2A). Histopathological characteristics of the colon and metabolic characteristics of lamina propria immune cells in the colon were analyzed on day 46 (chronic colitis) and after a remission period on day 67 (chronic remission, Figure 2A). Histopathological analyses of HE stainings were performed using an established scoring system [26] that includes the infiltration of immune cells, epithelial damage and goblet cell loss, severity and the percentage of affected tissue (Table 1). Importantly, as a treatment control for the experimental mice suffering from acute and chronic colitis, we used mice of the same age as their experimental counterparts, but which received only water ad libitum instead of DSS.

### 3.3. Abnormal Mitochondrial Phenotype of Colon Lamina Propria CD4+ and CD8+ T Cells During DSS-Induced Chronic Colitis Is Resolved During the Remission Phase

Histopathological analyses of the colons showed that mice undergoing acute DSS colitis had a significant increase (mean score of 11.8) in the histopathology score compared to control mice (mean score 0) (Table 2 and Figure S1). Importantly, the remission phase of 19 days following acute DSS treatment mitigated the histopathological score to ~6 (mean score of 5.9). Mice in the chronic-DSS group showed a significantly increased histopathological score of ~13 (mean score of 13.3) compared to the control group (mean score 0), while mice following remission from chronic colitis had less severe colonic inflammation (mean score 7.8).

Transcriptomic analyses have shown a significant alteration of mitochondrial gene expression in total rectal biopsies of UC patients (Figure 2B,C) [23]. In order to study the metabolic characteristics of adaptive and innate immune cells following acute and chronic DSS colitis, as well as during the respective remission phases, we isolated lamina propria leukocytes and IECs from colons of the indicated timepoints and groups followed by flow cytometry analyses. Specifically, we analyzed cellular mROS production using Mitosox staining and mitochondrial size by means of Mitotracker staining of CD4+ T cells, CD8+ T cells, B cells, eosinophils, neutrophils and vital CD45-CD31-EpCAM+ intestinal epithelial cells. During acute colitis and the respective remission phase, CD4+ and CD8+ T cells from the colon lamina propria did not show alterations in mROS production and their mitochondrial size (Figure 2D,E). In contrast, during chronic DSS colitis, intestinal CD8+ T cells produced significantly less mROS, which was also accompanied by significantly reduced mitochondrial size compared to CD8+ T cells from the control mice. Importantly, reduced mROS production and mitochondrial size in ex vivo-analyzed colonic CD8+ cells were completely restored during the remission phase following chronic colitis. Similarly, CD4+ T cells showed a significant decrease in mitochondrial size following chronic DSS colitis, which was also resolved during remission (Figure 2E). In contrast, eosinophils, neutrophils, B cells and intestinal epithelial cells showed a trend of increased mROS production and mitochondrial size during remission compared to the chronic-DSS-colitis phase, which, however, did not reach statistical significance (Figure S2). Importantly, the observed reduction in the mitochondrial size in the DSS-induced chronic colon inflammation may be indicative of a reduced cellular ATP-production. To test whether reduced oxidative phosphorylation also mitigates mitochondrial size, we performed T cell stimulation assays in vitro with Oligomycin as a specific inhibitor of the ATP synthase. Oligomycin blocks the F_0_ subunit of the ATP synthase proton channel, which is required for oxidative phosphorylation of ADP to ATP. We stimulated primary murine CD4+ T cells with agonistic anti-CD3/anti-CD28 antibodies in vitro to boost cellular metabolic activity in the absence or presence of Oligomycin. Oligomycin significantly reduced the mitochondrial size in mitogen-stimulated CD4+ T cells (Figure 2F); however, the sample size was limited (*n* = 3). Together, our data show that the abnormal mitochondrial phenotype of CD4^+^ and CD8^+^ T cells in the colon during chronic DSS-induced colitis is resolved during the remission phase.

### 3.4. CD4+ and CD8+ T Cells Show Increased Glucose Uptake During Acute but Defective Glucose Consumption During Chronic DSS Colitis That Persists During Remission

**Figure 3:**
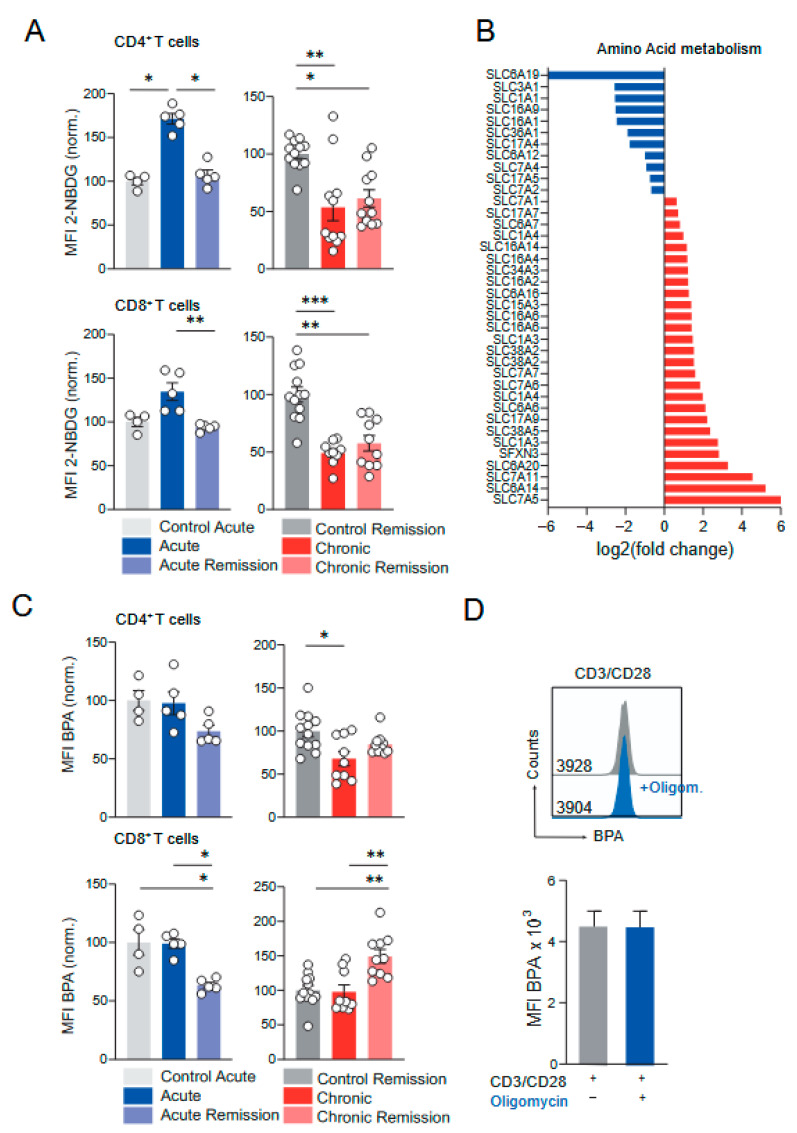
Glucose and amino acid uptake of T cells with mitochondrial depression from different stages of DSS-induced colitis. (**A**,**C**) Mice from the six different experimental groups of the DSS model were treated and sacrificed as denoted in Figure 2A. *Lamina propria* leukocytes were isolated using enzymatic tissue digestion followed by density-gradient centrifugation. (**A**). Glucose uptake was determined by 2-NBDG assay. For cell subset identification, cells were surface stained with lineage antibodies and subsequently analyzed by flow cytometry. Gated CD4^+^ and CD8^+^ T cells, were analyzed for their MFI of 2-NBDG fluorescence intensity. (**B**). Re-analysis of RNAseq data from human rectal biopsies of IBD patients. Identification of altered up- (red) and downregulated (blue) genes related to amino acid metabolism visualized as log_2_ (fold change). (**C**). Amino acid uptake was determined using BPA assay followed by cells lineage antibody staining and flow cytometric data acquisition. Gated CD4^+^ and CD8^+^ T cells were analyzed for their MFI of BPA fluorescence intensity. (**D**) Mixed leukocytes from spleen and lymph nodes (cervical, submandibular, brachial, axillary, inguinal, mesenteric) were isolated from untreated wild-type mice, and CD4^+^ T cells were isolated by magnetic negative selection. Cells were stimulated by agonistic CD3/CD28 (1 µg/mL each) antibodies in the absence and presence of 3 µM Oligomycin for three days. Aminoacid uptake was determined by BPA and measured by flow cytometry for the indicated groups MFI. Left: Representative histogram overlay. Numbers indicate MFI of histogram data. Right: Barplot showing mean ± SEM. Data in (**A**,**C**) for young control animals (*n* = 4, respectively, *n* = 5 for IECs) and the stages of acute colitis (*n* = 5) and remission of acute colitis (*n* = 5) are from one experiment (N = 1). Data from old control animals (*n* = 12) and the stages of chronic colitis (*n* = 11) and remission of chronic colitis (*n* = 11) are from two experiments (N = 2). Dots represent individual mice used to prepare cell solutions of the indicated cell subset. Statistical analysis was performed by the Kruskal–Wallis test (H-test) using values normalized to the mean of young and old control animals. * *p*  <  0.05, ** *p*  <  0.01, *** *p*  <  0.001. (**D**) Data is from one experiment (N = 1) using two individual mice (*n* = 2) to isolate and stimulate naïve CD4+ T cells.

During chronic colitis, CD4+ and CD8+ T cells showed an abnormal mitochondrial phenotype which was restored during remission. To test whether CD4+ and CD8+ T cells are metabolically affected in their ability to consume glucose to perform aerobic glycolysis during acute and chronic colitis, we measured their uptake of 2-(7-Nitro-2, 1, 3-benzoxadiazol-4-yl)-D-glucosamine (2-NBDG), a traceable fluorescent derivative of glucose. Interestingly, during acute colitis, colonic CD4+ and CD8+ T cells showed higher glucose consumption, which went back to baseline levels during the remission phase (Figure 3A). In contrast, the glucose consumption of CD4+ and CD8+ T cells was significantly reduced during chronic DSS colitis and did not normalize upon disease remission. Notably, similar to CD4+ and CD8+ T cells, B cells also showed a significant increase in glucose uptake during acute colitis, which returned to baseline levels during remission (Figure S3). In addition, during chronic colitis, neutrophils showed a significantly reduced glucose uptake that was reversed during the remission phase (Figure S3).

RNA sequencing of human rectal biopsies from UC patients revealed an alteration of genes encoding for solute carriers (SLCs) that transport amino acids—another cellular metabolic property (Figure 3B). However, the amino acid transporter activity of CD4+ and CD8+ T cells (Figure 3C) and eosinophils and neutrophils (Figure S4), analyzed by using Boronophenylalanine (BPA) staining and flow cytometry, was not significantly altered during acute and chronic colitis. In line with this, CD3/CD28-stimulated CD4+ T cells did show a normal amino acid uptake in the presence of Oligomycin (Figure 3D). In summary, lamina propria CD4+ and CD8+ T cells from the colon show increased glucose uptake during acute but defective glucose consumption during chronic DSS colitis, a cellular adaptation that is not restored during remission (Table 3).

## 4. Discussion

IBD, which includes UC and CD, is a global disease with increasing incidence, particularly in newly industrialized and developing countries [27,28]. Epidemiologic studies proved IBD to be a heterogeneous disease, with complex pathophysiology and diverse disease progression scenarios. Consequently, research in this field has led to a wide variety of treatment strategies to provide appropriate case-specific therapy options. However, new approaches are needed as many patients remain refractory to treatment [29]. The current paper builds on the efforts and insights of Haberman et al., 2019 [23], who provided evidence for metabolic alterations within rectal biopsy samples from patients with UC. Amongst other things, they observed a downregulation of mitochondrial gene expression during active UC. Here, we report that specifically CD4+ and CD8+ T cells in the colon produce significantly less mitochondrial reactive oxygen species and have smaller mitochondria during chronic DSS-induced colitis in mice, a phenotype which is resolved during remission. In addition, CD4+ and CD8+ T cells exhibit increased glucose uptake during acute but defective glucose consumption during chronic DSS colitis that does not normalize during the remission phase. For this, we speculate that prolonged activation of CD4+ T cells may lead to metabolic exhaustion—characterized by reduced glucose consumption—which could result from overstimulation or nutrient deprivation over time.

Our findings highlight the dysfunction and the involvement of mitochondria within T cells during the pathogenesis of IBD as we detect significantly reduced mROS production and decreased mitochondrial volume of CD4^+^ and CD8^+^ T cells during chronic colonic inflammation, which return to baseline levels during remission. However, this finding does not implicate that overall cellular function restores during remission of the chronic state. Our observations are in line with other studies reporting mitochondrial dysfunction to be implicated in IBD [23,30,31,32]. In this context, directly modulating mitochondrial metabolism in T cells using approved drugs or novel small-molecule inhibitors like fenofibrate or carnitine-derived molecules, which provide alternative mitochondrial energy sources by enhancing fatty acid oxidation, resvetrarol, a PGC-1α modulator which enhances mitochondrial biogenesis, or the Drp1 inhibitor Mdivi-1, which prevents excessive mitochondrial fission, may account for novel strategies in IBD treatment [33,34,35].

Furthermore, as mitochondria derive from bacteria [36], one could postulate their direct communication with the gut microbiota. Microbiota are capable of directly influencing host ROS production and mitochondrial host homeostasis while, vice versa, increased ROS levels influence microbiota balance and can lead to a dysbiosis that is associated with IBD [37,38,39,40]. In line with this, DSS-induced colitis was recently reported to be accompanied by transient changes in the microbiota composition [26]. Hence, the microbiota–mitochondria axis could be an interesting target for novel therapeutic approaches [41]. However, if and how T cell-derived mROS may affect these mechanisms remains unclear and is subject to further investigation.

The acute-DSS group in our mouse model reflects the first colon inflammatory episode with a loss of the intestinal barrier. Hence, in this period of our model, a plethora of microbial and food antigens trigger a multitude of new T cell responses accompanied by substantial T cell activation. Fulminant T cell stimulation depends on glucose uptake and glycolytic capacity. Thus, enhanced glucose uptake of CD4+ and CD8+ T cells in the acute-DSS group may be interpreted as a proxy for T cell activation. Indeed, T cells switch from mitochondria-dependent oxidative phosphorylation (OXPHOS) to aerobic glycolysis upon activation in order to allow for metabolically expensive cell proliferation. Inversely, the latter scenario could also explain the observed normalization of the glucose uptake capacity during the acute remission phase, accompanied by declining T cell activation processes due to the re-established barrier function of the colon. This leaves behind a plethora of antigen-experienced yet quiescent lamina propria memory T cells with a low rate of metabolism. Together, our findings here reveal an atypical mitochondrial phenotype of colonic CD4+ and CD8+ T cells during chronic colitis that is restored during remission.

However, our study also has limitations. Firstly, we do not measure the mitochondrial function in real-time but determine surrogate parameters, including mROS production and mitochondrial volume. Secondly, we do not account for the plasticity of CD4+ T cells, which differentiate into various Th cell populations that take part in the pathophysiology of IBD. Additionally, it is not fully understood to what extent our mouse model replicates human IBD, nor are the possible influences of age and gender differences (only female mice used) on disease development and response taken into account. For the reanalysis of human transcriptomic data, there is no categorization of patients based on their disease severity or treatment status, which may potentially influence the findings. Lastly, all mice were the same age at the start of the experiment (11 weeks) but sampled at different times, which means that age-related differences cannot be entirely excluded, especially for chronic-phase results. Addressing these limitations in future research will help strengthen the translational relevance of our findings.

## Figures and Tables

**Table 1:** Histological scoring [26].

Histological Changes	Score: 0	Score: 1	Score: 2	Score: 3	Score: 4
Infiltration of immune cells	No inflammation	Around crypt base	Into mucosa	Extensive mucosal infiltration and edema	Into submucosa
Epithelial damage and loss of goblet cells	Intact	Slight loss of goblet cells	Considerable loss of goblet cells and slight loss of intestinal crypts	Extensive loss of intestinal crypts	
Extent	None	Mucosa	Mucosa and submucosa	Transmural	
Percent involvement		1–25%	26–50%	51–75%	76–100%

**Table 2:** Histopathology Score DSS colitis.

Conditions	Day	Histopathological Score	Adj. *p*-Value (Control Group)	% Tissue with Colon Inflammation	Adj. *p*-Value (Control Group)
Control young	11	0		0	
Acute	6	11.8	0.0018 (**)	1.8	0.0596 (ns)
Remission acute	25	5.9	>0.9999 (ns)	1.0	>0.9999 (ns)
Control old	56	0		0	
Chronic	46	13.3	<0.0001 (****)	3.3	<0.0001 (****)
Remission chronic	67	7.8	0.1845 (ns)	3	0.0003 (***)

The table presents the histopathology scores, percentage of tissue with colon inflammation, and corresponding adjusted *p*-values (adj. *p*-value) compared to control groups. The control young mice serve as the control group for the acute model and control old mice for the chronic model. Data are shown for young and old control mice, acute, remission after acute, chronic and remission after chronic-DSS treatment. The scores reflect the severity of tissue damage and inflammation, with higher values indicating more severe pathology based on Table 1. Histological data were collected from two separate experiments for all DSS treatment groups (*n* = 8), except for the acute-DSS colitis group, which was derived from three independent experiments (*n* = 10), and the control groups, which came from a single experiment (*n* = 6). The analysis was conducted in a blinded manner. Significance calculated using the Kruskal–Wallis test. Statistical significance is indicated by adj. *p* value and with the following categorization: ** *p*  <  0.01, *** *p*  <  0.001, **** *p* < 0.0001, and ns denotes non-significant differences relative to controls.

**Table 3:** Summary of alterations in mitochondrial ROS production, mitochondrial volume and glucose consumption in immune cell subsets during DSS-induced colitis.

	Acute Colitis		Remission Acute		Chronic Colitis		Remission Chronic	
**Mitochondrial ROS production**	CD4+ T	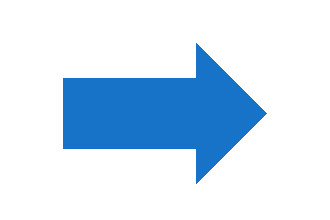	CD4+ T	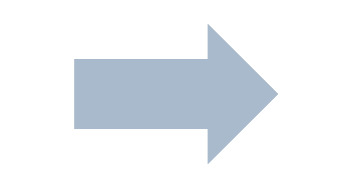	CD4+ T	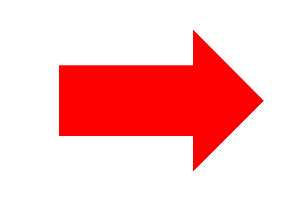	CD4+ T	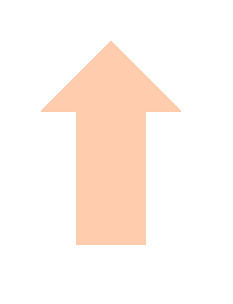
	CD8+ T	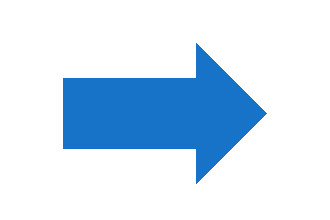	CD8+ T	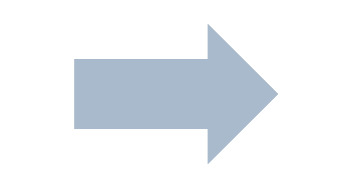	CD8+ T	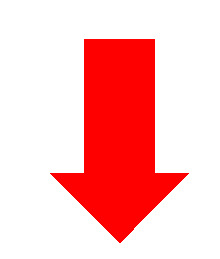	CD8+ T	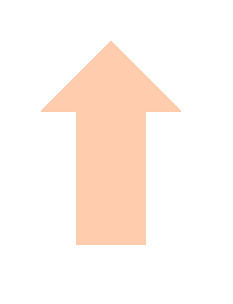
	B	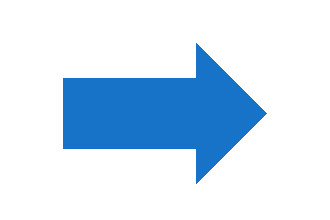	B	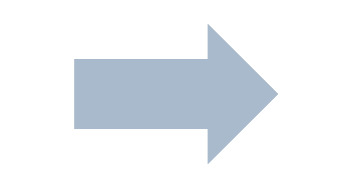	B	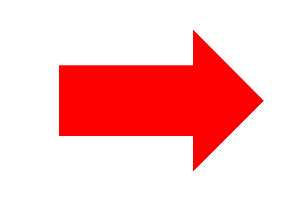	B	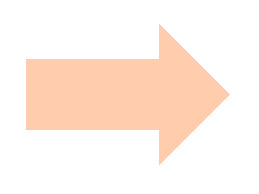
	Neut.	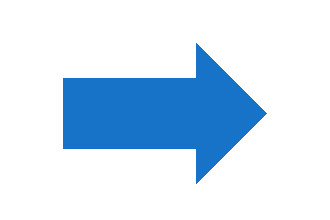	Neut.	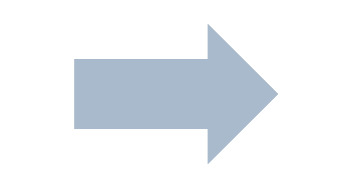	Neut.	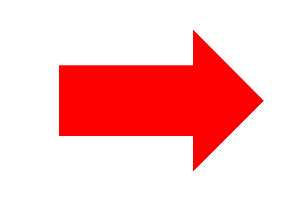	Neut.	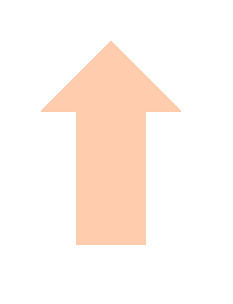
	Eos.	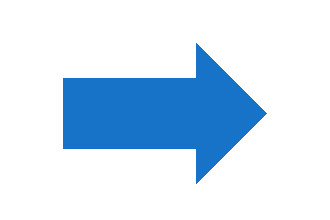	Eos.	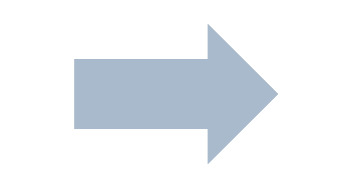	Eos.	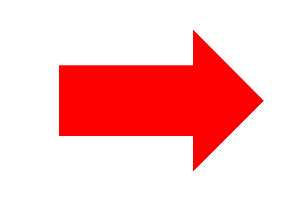	Eos.	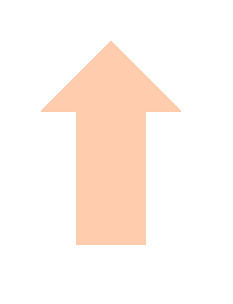
**Mitochondrial volume**	CD4+ T	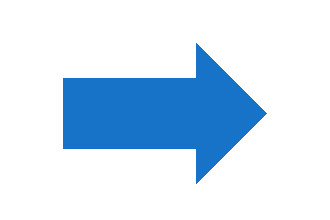	CD4+ T	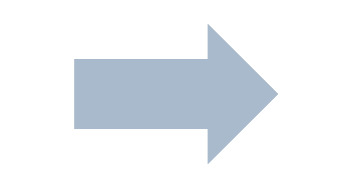	CD4+ T	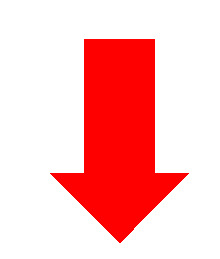	CD4+ T	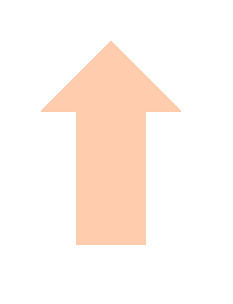
	CD8+ T	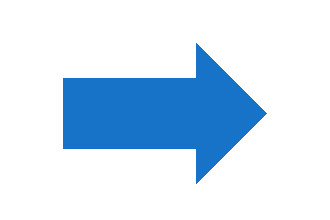	CD8+ T	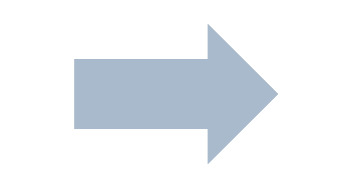	CD8+ T	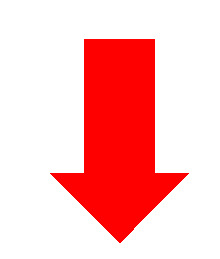	CD8+ T	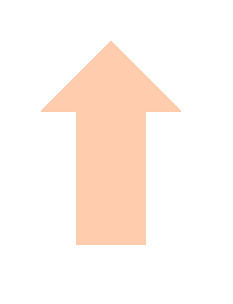
	B	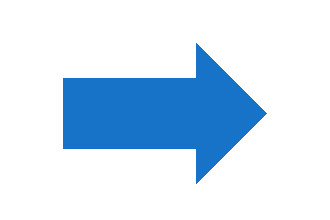	B	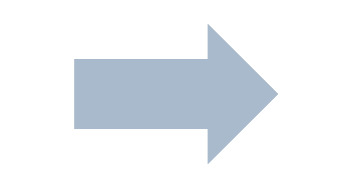	B	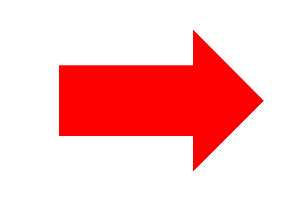	B	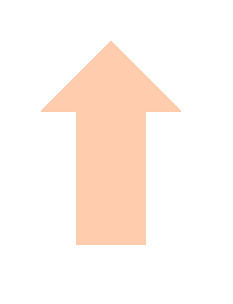
	Neut.	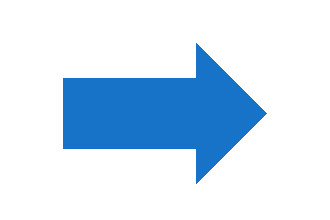	Neut.	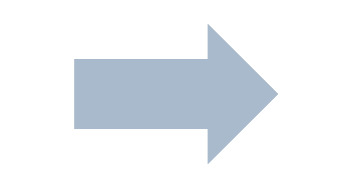	Neut.	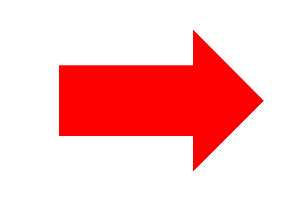	Neut.	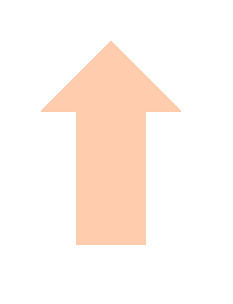
	Eos.	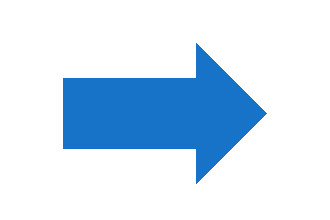	Eos.	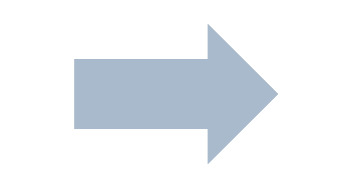	Eos.	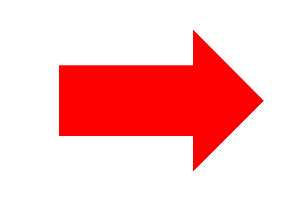	Eos.	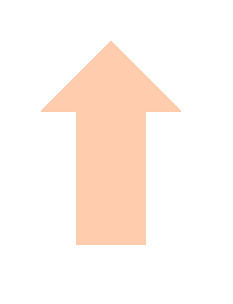
**Glucose consumption**	CD4+ T	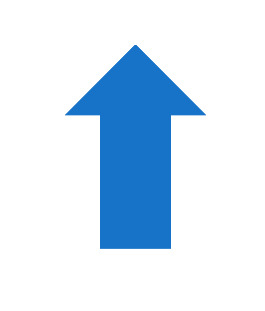	CD4+ T	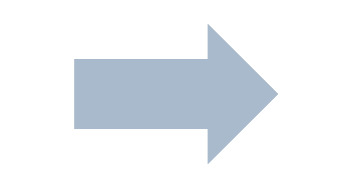	CD4+ T	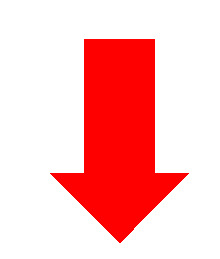	CD4+ T	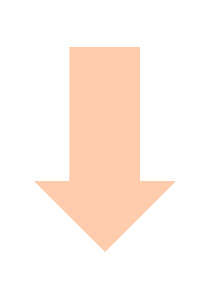
	CD8+ T	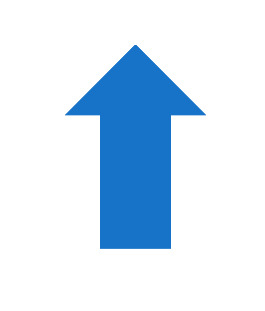	CD8+ T	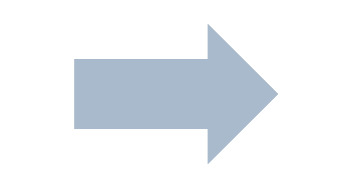	CD8+ T	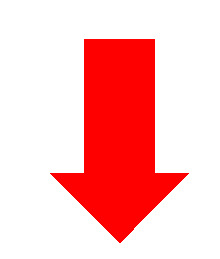	CD8+ T	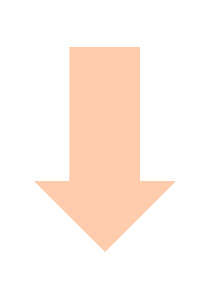
	B	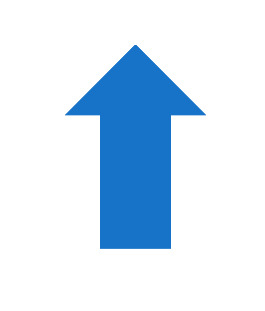	B	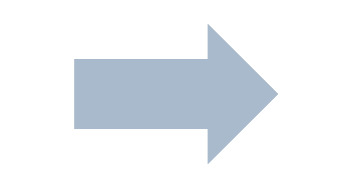	B	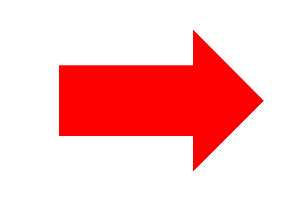	B	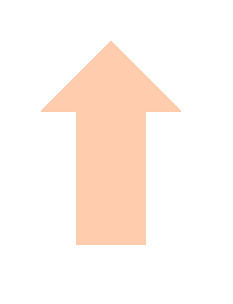
	Neut.	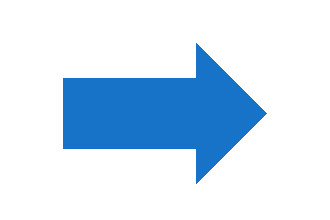	Neut.	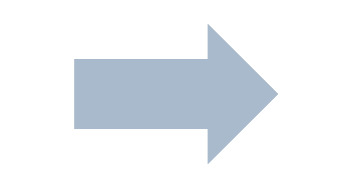	Neut.	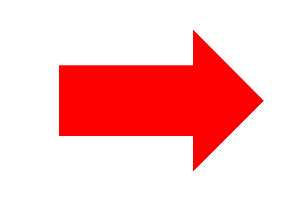	Neut.	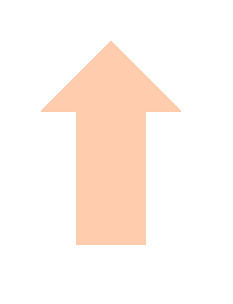
	Eos.	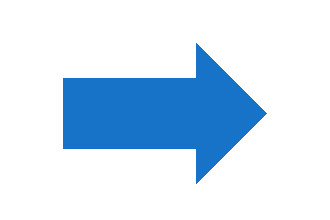	Eos.	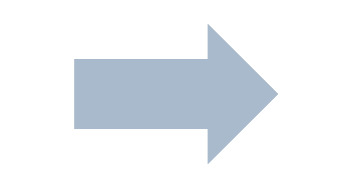	Eos.	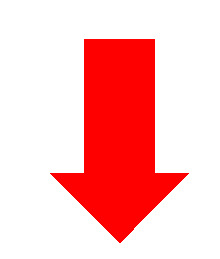	Eos.	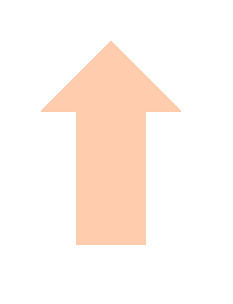

Arrows indicate the direction of change relative to healthy controls: ↑, increase; ↓, decrease; →, no change. Blue arrows represent changes during acute colitis and its remission; red arrows represent chronic colitis; light-red arrows represent remission after chronic colitis. Immune cell subsets analyzed: CD4^+^ T cells, CD8^+^ T cells, B cells, neutrophils (Neut.), and eosinophils (Eos.). All measurements were performed by flow cytometry: mitochondrial ROS production was determined by Mitosox, mitochondrial volume by Mitotracker, and glucose consumption by 2-NBDG uptake. Data represent the indicated immune cell subsets across the different disease stages in DSS-induced inflammatory bowel disease (IBD).

## Data Availability

PROTECT (https://www.ncbi.nlm.nih.gov/geo/query/acc.cgi?acc=GSE109142, accessed on 14 January 2025) and RISK (https://www.ncbi.nlm.nih.gov/geo/query/acc.cgi?acc=GSE117993, accessed on 14 January 2025) rectal mRNAseq data sets were published in [23] and deposited in GEO. The FACS data that support the findings of this study are available from the authors of this study upon reasonable request.

## Supplementary Materials

The following supporting information can be downloaded at https://www.mdpi.com/article/10.3390/biomedicines13092094/s1, Figure S1: Representative histological images of the colon tissue, stained with H&E for the indicated groups (scale bar: 100 μm); Figure S2: A. Re-analysis of RNAseq data from human rectal biopsies of IBD patients. Identification of altered genes related to Hematopoetic stem cells visualized as log2(fold change) using KEGG pathway analysis. mROS production levels determined by Mitosox and measured by FACS in eosinophils, and neutrophils (B) as well as B cells (D) and intestinal epithelial cells (F) during the indicated stages of DSS colitis. Mitochondrial size de-termined by Mitotracker and measured by FACS in eosinophils, and neutrophils (C) as well as B cells (E) and intestinal epithelial cells (G) during the indicated stages of DSS colitis. B-G. All data of young control animals (*n* = 4, respectively *n* = 5 for IECs), and the stages of acute colitis (*n* = 5) and remission of acute colitis (*n* = 5) are from one experiment (N = 1). Data from old control animals (*n* = 12), and the stages of chronic colitis (*n* = 11) and remission of chronic colitis (*n* = 11) are from two experiments (N = 2).; Figure S3: A. Glucose consumption determined by 2-NBDG measured by FACS in eosinophils, and neutrophils (A) as well as B cells (B) and intestinal epithelial cells (C) during acute and chronic DSS colitis as well as remission; Figure S4: A. Amino acid uptake was determined using BPA assay in eosinophils, and neutrophils during acute and chronic DSS colitis as well as remission. Cells were analyzed for their MFI of BPA fluorescence intensity using FACS. Frequencies of CD36+ B cells (B) and intestinal epithelial cells (C) during acute and chronic DSS colitis as well as remission measured by flow cytometry.
