# Supplementary material for: Atypical Mitochondrial Phenotype of Colonic CD4+ and CD8+ T Cells During Experimental Chronic Colitis
Source: Biomedicines. 2025 Aug 28;13(9):2094. doi: 10.3390/biomedicines13092094 (PMC12467582; doi:10.3390/biomedicines13092094)
Supplement: Supplementary file 1 [file biomedicines-13-02094-s001.zip › biomedicines-3721814-supplementary.pdf]

biomedicines

MDPI

Supplementary information

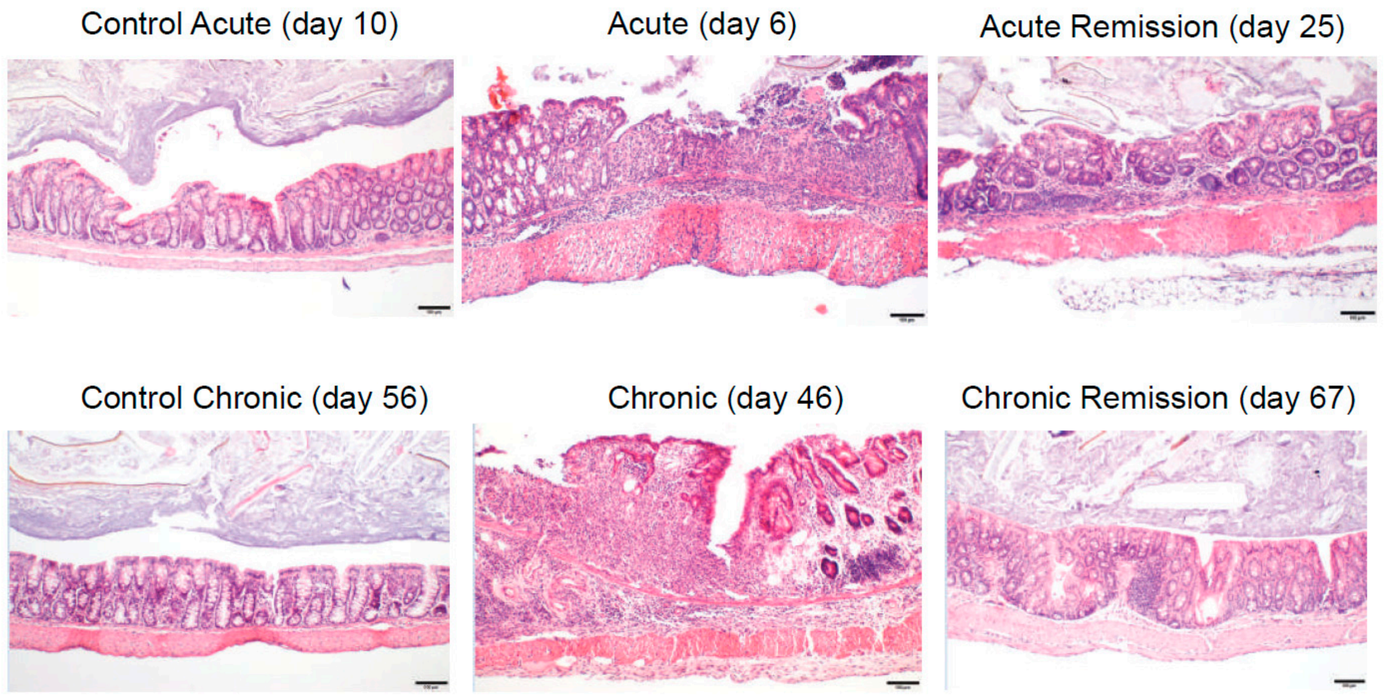

Figure.S1: Representative histological images of the colon tissue, stained with H&E for the indicated groups (scale bar: 100 μm).

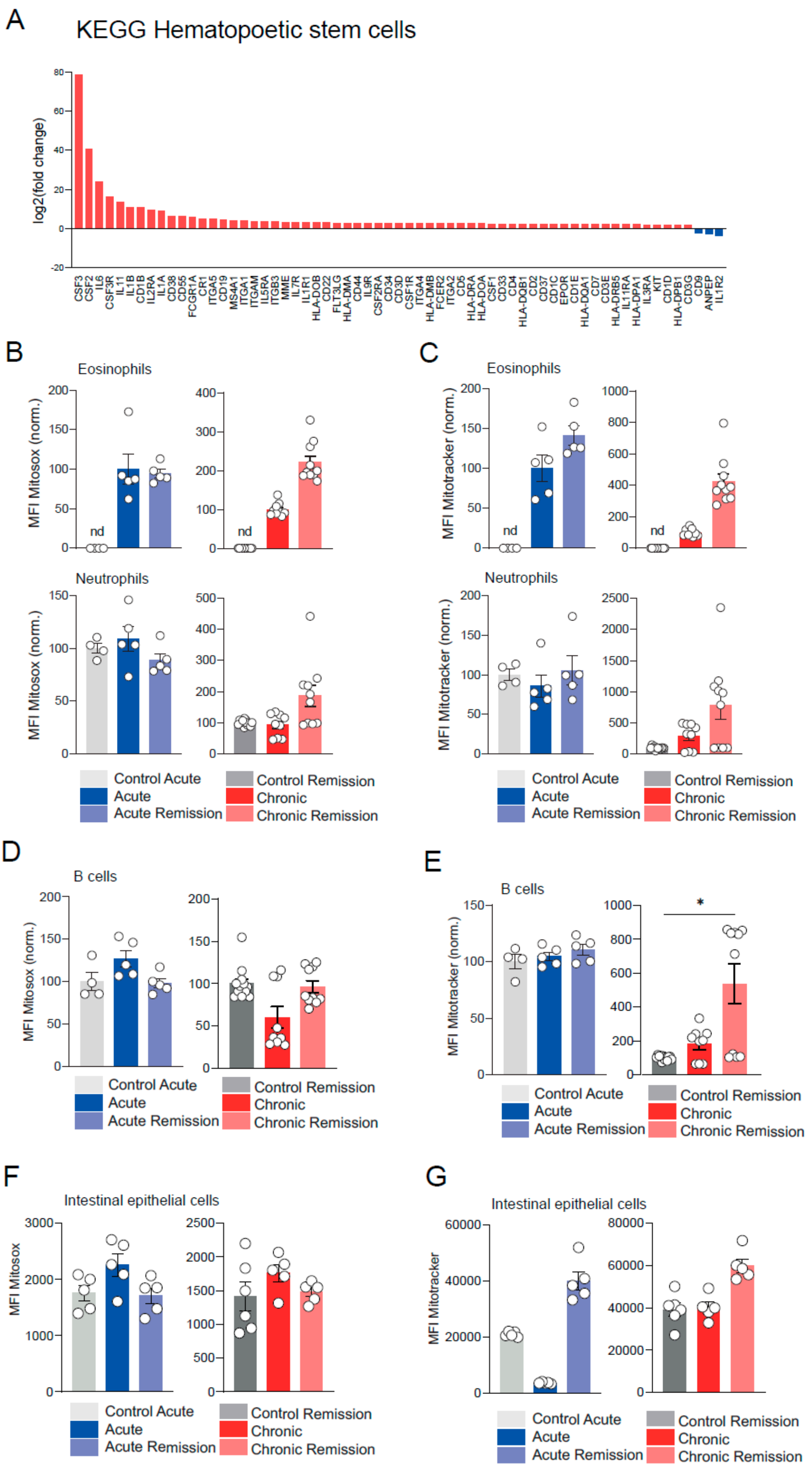

Figure. S2: A. Re-analysis of RNAseq data from human rectal biopsies of IBD patients. Identification of altered genes related to Hematopoetic stem cells visualized as log_2(fold change) using KEGG pathway analysis. mROS production levels determined by Mitosox and measured by FACS in eosinophils, and neutrophils (**B**) as well as B cells (**D**) and intestinal epithelial cells (**F**) during the indicated stages of DSS colitis. Mitochondrial size determined by Mitotracker and measured by FACS

in eosinophils, and neutrophils (**C**) as well as B cells (**E**) and intestinal epithelial cells (**G**) during the indicated stages of DSS colitis. B-G. All data of young control animals (n = 4, respectively n = 5 for IECs), and the stages of acute colitis (n = 5) and remission of acute colitis (n = 5) are from one experiment (N = 1). Data from old control animals (n = 12), and the stages of chronic colitis (n = 11) and remission of chronic colitis (n = 11) are from two experiments (N = 2).

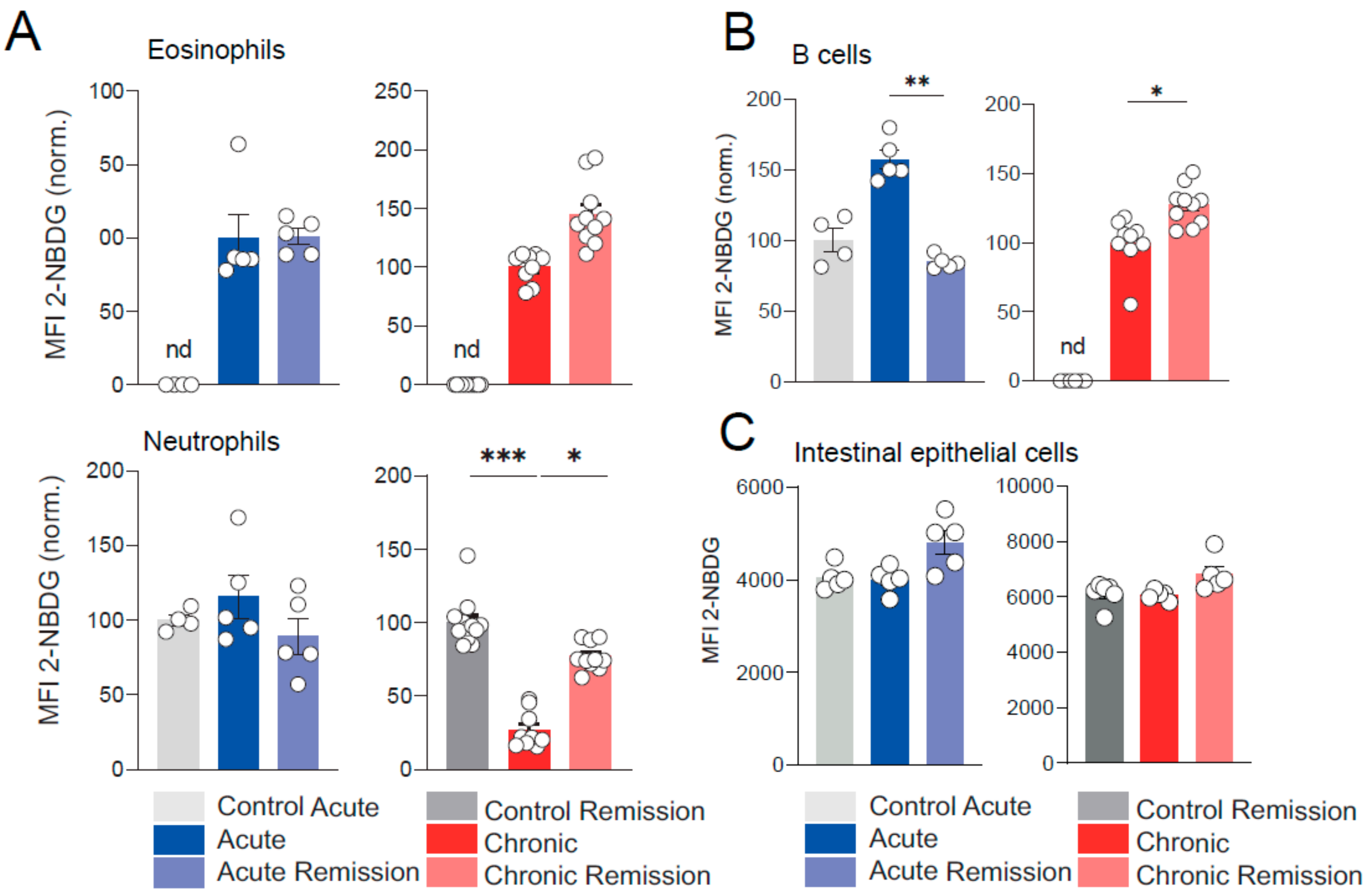

Figure S3. A. Glucose consumption determined by 2-NBDG measured by FACS in eosinophils, and neutrophils (**A**) as well as B cells (**B**) and intestinal epithelial cells (**C**) during acute and chronic DSS colitis as well as remission.

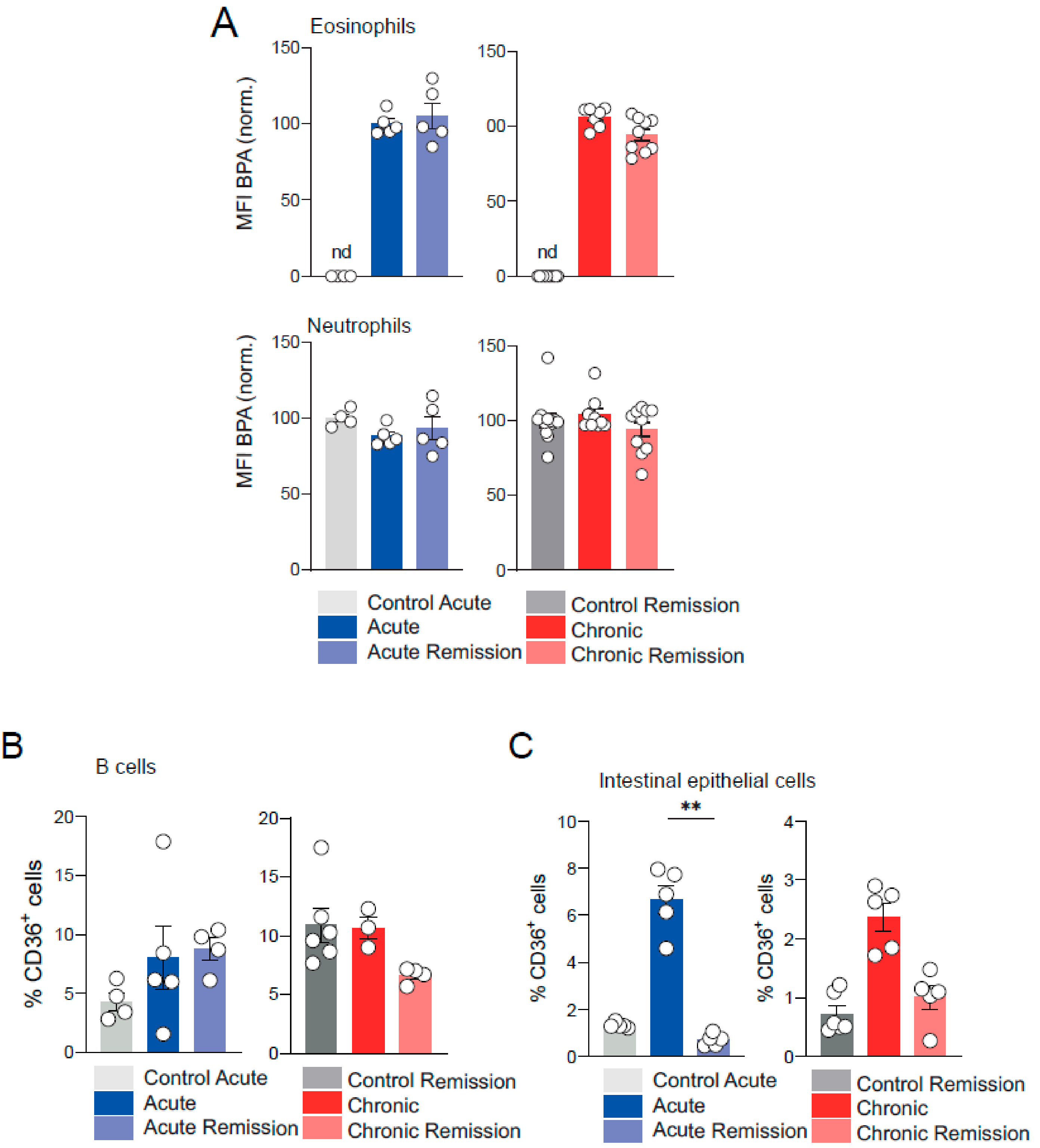

Figure S4. A. Amino acid uptake was determined using BPA assay in eosinophils, and neutrophils during acute and chronic DSS colitis as well as remission. Cells were analyzed for their MFI of BPA fluorescence intensity using FACS. Frequencies of CD36+ B cells (**B**) and intestinal epithelial cells (**C**) during acute and chronic DSS colitis as well as remission measured by flow cytometry.
